# Acoustic allometry revisited: morphological determinants of fundamental frequency in primate vocal production

**DOI:** 10.1038/s41598-017-11000-x

**Published:** 2017-09-05

**Authors:** Maxime Garcia, Christian T. Herbst, Daniel L. Bowling, Jacob C. Dunn, W. Tecumseh Fitch

**Affiliations:** 1 Department of Cognitive Biology, University of Vienna, Althanstrasse 14, 1090 Vienna, Austria; 2 ENES Lab, Université Lyon/Saint-Etienne, NEURO-PSI, CNRS UMR 9197 Saint-Etienne, France; 3 Division of Biological Anthropology, University of Cambridge, Pembroke Street, Cambridge, CB2 3QG UK; 4 Animal and Environment Research Group, Anglia Ruskin University, East Road, Cambridge, CB1 1PT UK

**Keywords:** Behavioural ecology, Evolutionary ecology, Biomechanics

## Abstract

A fundamental issue in the evolution of communication is the degree to which signals convey accurate (“honest”) information about the signaler. In bioacoustics, the assumption that fundamental frequency (*f*_o_) should correlate with the body size of the caller is widespread, but this belief has been challenged by various studies, possibly because larynx size and body size can vary independently. In the present comparative study, we conducted excised larynx experiments to investigate this hypothesis rigorously and explore the determinants of *f*_o_. Using specimens from eleven primate species, we carried out an inter-specific investigation, examining correlations between the minimum *f*_o_ produced by the sound source, body size and vocal fold length (VFL). We found that, across species, VFL predicted minimum *f*_o_ much better than body size, clearly demonstrating the potential for decoupling between larynx size and body size in primates. These findings shed new light on the diversity of primate vocalizations and vocal morphology, highlighting the importance of vocal physiology in understanding the evolution of mammal vocal communication.

## Introduction

The question of when and why animal signals convey accurate information about the signaler or environment – the problem of “honest” communication – has a long history^1–3^. In the domain of acoustic communication, important insights have recently come from applying a better understanding of the vocal production mechanism to this issue^4–7^. These studies indicate that increased understanding of the signal production mechanism can play a central role in explaining what components of a signal convey honest information, and why (e.g. ref.^8^).

Contemporary understanding of vocal production in mammal communication has benefited greatly from adopting the source-filter theory of vocal production^4,5,9,10^. According to this framework^11^, originally developed for human speech and later applied to animal communication, a sound is produced by the vibrating vocal folds within the larynx (the sound source) and their vibration rate determines the fundamental frequency (hereafter *f*_o_) of the acoustic signal. This source signal then propagates through the vocal tract, where airborne resonances (which vary with vocal tract length and shape) emphasize some frequencies, called formants.

The connection between the acoustic characteristics of vocalizations and the physical attributes of the signaler suggests that key aspects of sound production can be anatomically constrained, with much research focusing on the relationship between a caller’s body size and *f*_o_^7,12,13^. In particular, Morton^12^ postulated that an animal’s body size should be negatively related to the frequency content of its voice (including *f*_o_), although he did not specify the physical and/or physiological factors that would underlie this putative correlation.

The prediction of a correlation between body size and *f*_o_ relies on two main assumptions^5^: 1) body size directly determines the size of the larynx and therefore, the length of the vocal folds (as vocal folds in mammalian larynges extend from the thyroid cartilage to the arytenoids^14^), and 2) that the resting (i.e., unstretched) vocal fold length (hereafter VFL) has a direct influence on *f*_o_. Biomechanical theory corroborates the latter condition, predicting that longer focal folds produce lower *f*_o_^15^. However, the former assumption has been challenged, given that larynx size is not necessarily constrained by body size^5,8^. Indeed various intraspecific studies, in multiple species, have failed to reveal the expected size-frequency relationship, finding a weak or non-existent correlation between body size and *f*_o_ within adults of a given species^8,16–19^.

Research on primate vocal production has also followed this general line of thought regarding the body size − *f*_o_ relationship. A literature-based analysis conducted by Hauser^20^ concluded that ‘larger species produce relatively lower-pitched vocalizations than smaller species’, relying on an amalgam of various frequency measures determined by visual inspection of printed spectrograms. In Hauser’s study however, the methodology applied to designate ‘frequency’ pooled manual measurements of the dominant frequency (hereafter DF) and *f*_o_. The interpretation of Hauser’s results is difficult because *f*_o_ and DF reflect different acoustic phenomena: while *f*_o_ reflects the rate of vibration of the vocal folds, DF is defined as the frequency at which the radiated acoustic spectrum has its greatest amplitude (see e.g. ref.^21^). DF is influenced by both the spectral composition of the laryngeal sound source and the filtering characteristics of the vocal tract. Such a conflation of distinct causal factors could easily confound the quantitative estimation of the relationship between frequency and body size across species, as shown by a recent study conducted on a wide range of vocalizations from numerous primate and carnivore species^7^.

Another complicating factor is that *f*_o_ can strongly depend on several parameters besides VFL. For example, an increase in subglottal pressure (hereafter Psub), determined by the air pressure from the lungs, typically leads to an increase in *f*_o_^22,23^. Likewise, an increase in the tension applied to the vocal folds has similar effects: stretching of the vocal folds by the action of the cricothyroid muscle^15^ increases tension and stiffness, leading to a higher *f*_o_^24^. Vocal fold mass may also affect *f*_o_, although this has recently been disputed^25^. Finally, the vocal folds are multilayered structures^26^ and layer composition varies across species^27,28^, which could influence elasticity^29^ and thus *f*_o_^30^.

The influence of these multiple factors means that for a given VFL and tissue composition, an animal can in principle greatly increase *f*_o_ by increasing Psub^24^ and vocal fold tension^31^. Analyses of vocalizations produced by free-moving animals, including the study conducted by Hauser^20^ and the most recent large-scale analyses on the question^6,7^, cannot account for these confounding factors. The use of experimentally-controlled *in vitro* phonation in an excised larynx setup offers a major advantage in this respect, providing accurate measurement and control of key factors, such as Psub and vocal fold tension^32^. Unlike *in vivo* conditions, excised larynx experiments also allow us to adjust and precisely document laryngeal geometry and vocal fold position.

In the present comparative study we use an automated excised larynx setup to investigate larynges from 11 primate species, phonated in a controlled laboratory setting, to examine the physical and physiological determinants of inter-specific variation of primate *f*_o_ in detail. Our underlying physical model is given in equation (1)^15^, representing a simple string model of a vibrating vocal fold, where L is the VFL, σ is the tensile stress in the vocal fold and ρ the tissue density:

1 $${f}_{o}=\frac{1}{2L}\sqrt{\frac{\sigma }{2L}}[{\rm{Hz}}]$$

Equation (1) suggests that, given constant tissue density and VFL, the lowest *f*_o_ is reached at minimal tensile stress. This condition can easily be met in an excised larynx preparation where vocal folds can be adducted without being elongated. At this stage (fixed tissue density and minimal tension), VFL should be the key determinant of *f*_o_. Because *f*_o_ decreases with Psub^23,24^, the lowest attainable *f*_o_ should then occur at the lowest pressure inducing phonation, i.e. at phonation threshold pressure (hereafter PTP). Again, an excised larynx preparation allows this to be controlled by progressively adjusting pressure until reaching PTP, where *f*_o_ should be at a minimum and mainly dependent on the resting VFL. Thus, for a fixed tissue density and with minimal tension and Psub, measuring the minimum *f*_o_ (hereafter min*f*_o_) that a given larynx can produce is predicted by theory to be the most appropriate standardized approach to determine to what degree *f*_o_ provides an honest indicator of body size.

We investigated specifically how well min*f*_o_ and other *f*_o_ measures are predicted by both VFL and body size across species, using individual larynges from 11 different primate species. We carried out CT-scans of excised larynges from individuals of known body size, in order to obtain anatomical estimates of VFL for each specimen, and then phonated these same specimens in an excised larynx setup under controlled conditions of Psub and minimal vocal fold tension. Because larynx size and body size are not necessarily correlated, we predicted that VFL, rather than body size, should best predict the min*f*_o_ of acoustic signals. Although the decoupling of larynx size and body size has been previously discussed in primate vocal production^5,33^, the present study is the first empirical test of the physics underlying this prediction, using a controlled *in vitro* setup and matching anatomical and acoustical measurements from the same individuals. Based on the considerable diversity found both in primate vocal signals and vocal anatomy^5,34–36^ we discuss our results in the context of evolutionary pressures that may have influenced vocal production in primates and mammals more generally.

## Results

### Anatomical relationship between body size and VFL across species

**Figure 1:**
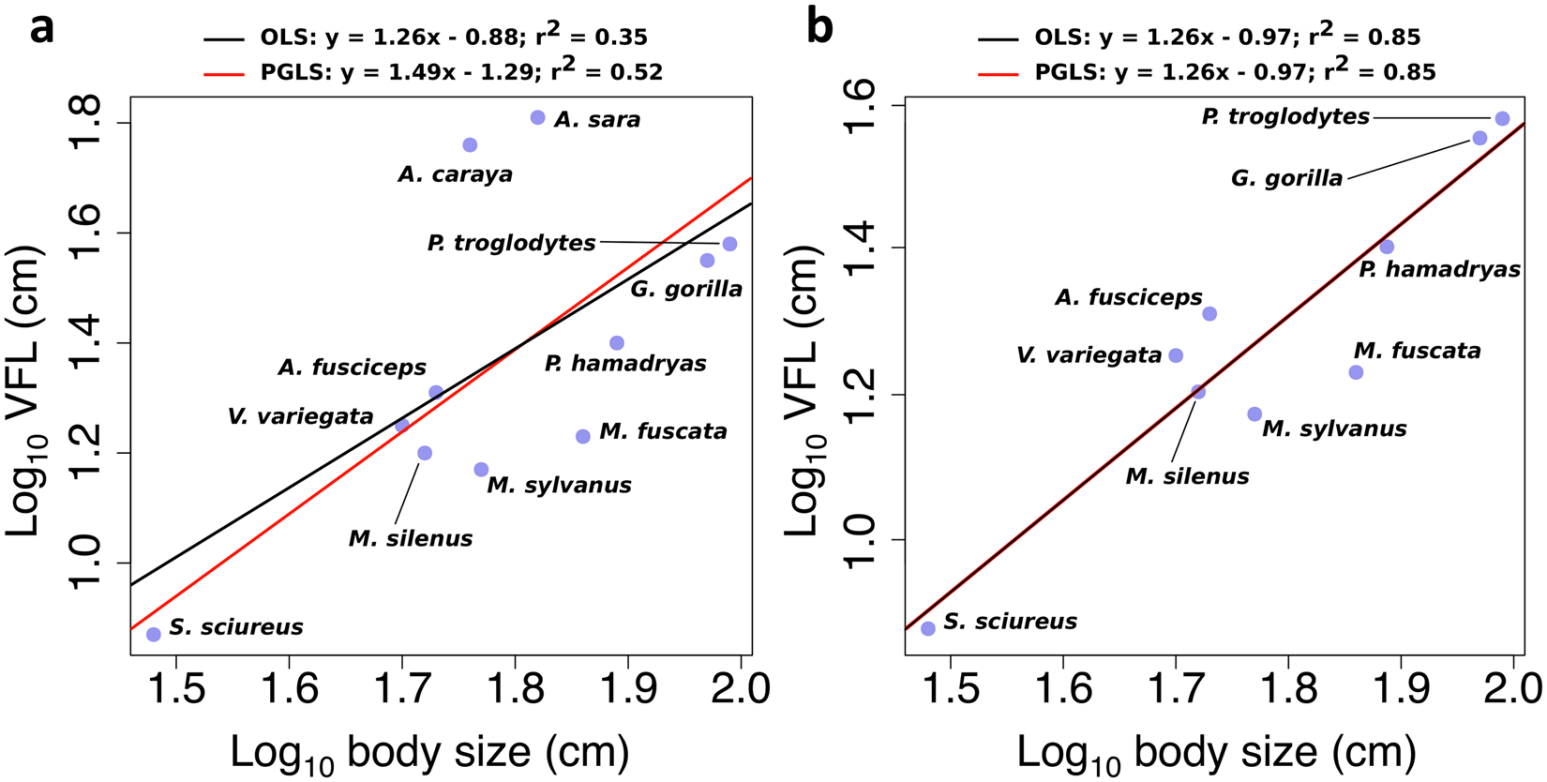
Decoupling of body size and vocal fold length. Bivariate plots illustrating the relationship between the base-10 logarithms of body size and VFL: (**a**) including howler species; (**b**) excluding howler species. Black lines depict OLS regressions and red lines depict PGLS regressions.

Ordinary least squares (OLS) regression showed a significant positive relationship between log VFL and log body size (r^2^ = 0.35, β = 1.26, t = 2.51, P = 0.03; Fig. 1a), which was confirmed by the phylogenetic generalized least squares (PGLS) regression (r^2^ = 0.52, λ = 1.00, t = 3.44, P = 0.007). Excluding howler monkeys (which can be suspected to be outliers in this type of regression given their highly enlarged vocal apparatus^37^) from the analysis (Fig. 1b) did not change the nature of the significant positive relationship (β = 1.26, t = 6.76, P < 0.001) but greatly improved the fit (OLS r^2^ = 0.85); PGLS regression was equivalent to OLS (λ = 0.00; Fig. 1b). This supports both the outlier status of howler species and the potential for decoupling between larynx size and body size across primate species.

### Acoustic allometry: Prediction of min*f*_o_ from body size and VFL across species

**Figure 2:**
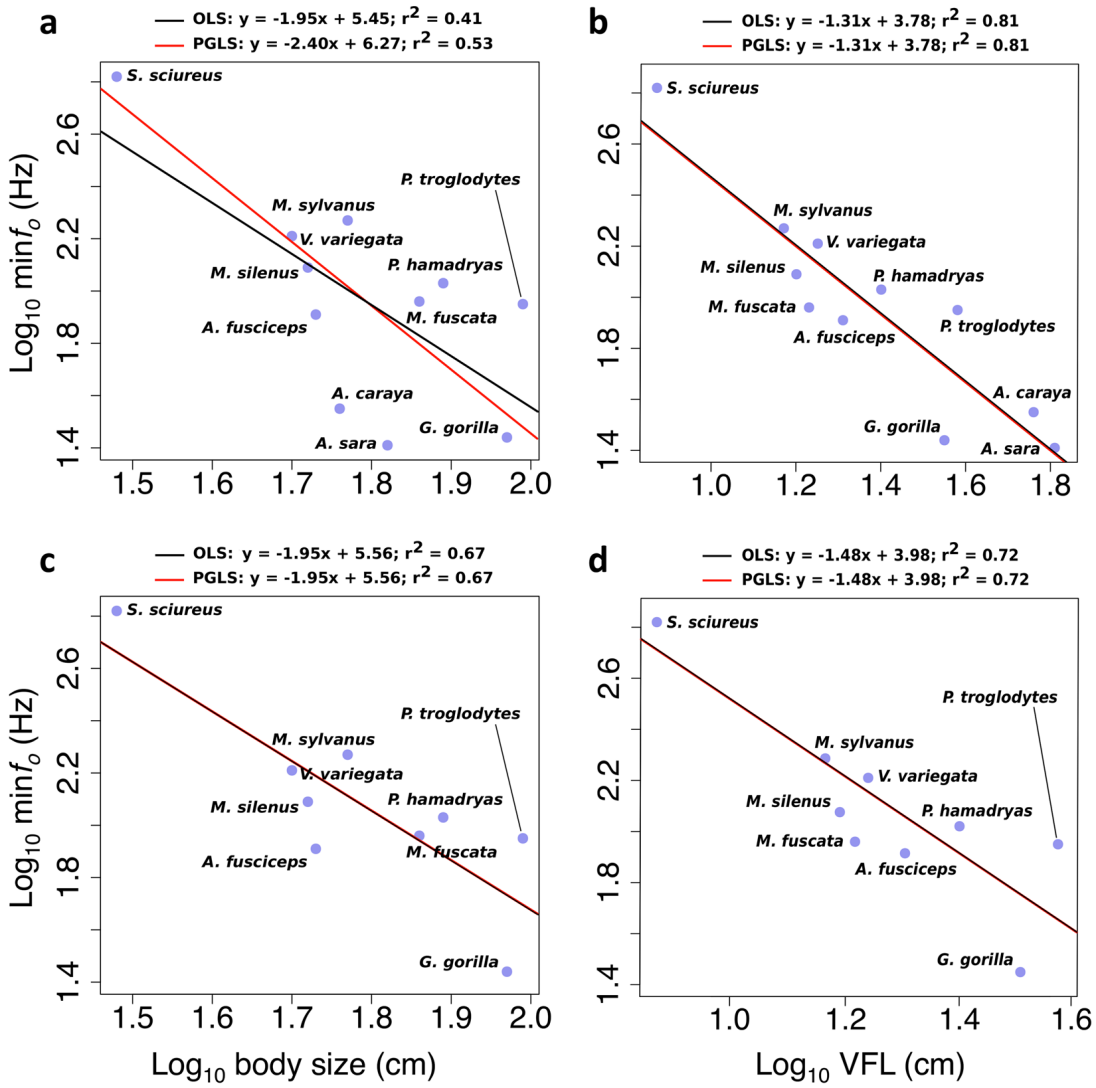
Acoustic allometry from primate laryngeal specimens. Bivariate plots illustrating relationships between the base-10 logarithms of (**a**) body size and min*f*_o_ and (**b**) VFL and min*f*_o_, for all 11 primate species considered here; (**c**,**d**) show the same relationships excluding the 2 howler monkey species. Black lines depict OLS regressions and red lines depict PGLS regressions.

Having found this potential for decoupling between larynx and body size, we then examined the inter-specific allometric relationship between these anatomical components and the acoustic production from the same specimens. OLS regressions indicated significant negative relationships for both log body size vs. log min*f*_o_ (β = −1.95, t = −2.82, P = 0.02; Fig. 2a) and log VFL vs. log min*f*_o_ (β = −1.31, t = −6.52, P < 0.001; Fig. 2b). Comparison of r-squared values suggest that log VFL was a much better predictor of min*f*_o_ than log body size (r^2^ = 0.81 vs. 0.41, respectively) (see Supplementary Table S2). The PGLS regressions supported these results, again showing significant negative relationships and that log VFL was a better predictor of min*f*_o_ than log body size (r^2^ = 0.81, λ = 0.00, t = −6.52, p < 0.001 and r^2^ = 0.53, λ = 0.59, t = −3.48, p = 0.007, respectively; Supplementary Table S2).

Repeating these analyses while excluding howler monkeys, similar results were obtained for both comparisons, with OLS regressions indicating significant negative relationships for both log body size vs. log min*f*_o_ (β = −1.95, t = −4.11, P = 0.004; Fig. 2c) and log VFL vs. log min*f*_o_ (β = −1.48, t = −4.64, P = 0.002; Fig. 2d). Once again prediction of min*f*_o_ by log VFL was stronger than by log body size (r^2^ = 0.72 vs. 0.67), although the difference was considerably reduced in comparison to the regression including howlers. This suggests that the inclusion of howlers is important for, but does not fully account for, the observed advantage of using VFL to predict min*f*_o_ compared to body size. The PGLS regressions excluding the two howler species were similar to their OLS counterparts, changing neither the fits nor significance levels (Supplementary Table S2).

### Acoustic allometry: Prediction of mean*f*_o_ and max*f*_o_ from body size and VFL across species

Parallel analyses were run using max*f*_o_ and mean*f*_o_ instead of min*f*_o_. As for log min*f*_o_, the OLS and PGLS regressions showed that both log mean*f*_o_ and log max*f*_o_ were better predicted by log VFL than by log body size (with all regressions being significant – all Ps ≤ 0.02). For the log VFL regressions, fits for log mean*f*_o_ and log max*f*_o_ (r^2^ = 0.7 and r^2^ = 0.74, respectively) were lower than for log min*f*_o_ (r^2^ = 0.81). PGLS regressions did not change these results. The same was not true for the body size regressions, where fits with log mean*f*_o_ and log max*f*_o_ (r^2^ = 0.46 and r^2^ = 0.43, respectively) were slightly higher than log min*f*_o_ (r^2^ = 0.41). PGLS regressions provided the same conclusions despite changing the fit of these models (r^2^ = 0.65, r^2^ = 0.56 and r^2^ = 0.53 for log mean*f*_o_, log max*f*_o_ and log min*f*_o_, respectively).

Excluding howler species, the results remained much the same, with OLS regressions showing better predictions for log VFL (mean*f*_o_ r^2^ = 0.67, max*f*_o_ r^2^ = 0.75) compared to log body size (mean*f*_o_ r^2^ = 0.6, max*f*_o_ r^2^ = 0.56). For the log VFL regressions, the fit for log mean*f*_o_ (r^2^ = 0.67) was lower than that for log min*f*_o_ (r^2^ = 0.72), itself lower than that for log max*f*_o_ (r^2^ = 0.75). However for the body size regressions, fits with log mean*f*_o_ and log max*f*_o_ (r^2^ = 0.6 and r^2^ = 0.56, respectively) were both lower than log min*f*_o_ (r^2^ = 0.67). PGLS regressions did not change any of the results from the analyses excluding howler species. See Supplementary Table S2 for full statistics on all of the above regressions. Inspection of the residual errors from the model including howler species confirmed our motive for running it again without howlers, as both species (*Alouatta caraya* and *Alouatta sara)* showed the highest absolute residuals in our regression.

### Driving pressure: Role of Psub in determining min*f*_o_

A Wilcoxon signed-rank test showed that the subglottal pressure at which min*f*_o_ is obtained (mean ± SE = 9.38 ± 0.94) was significantly lower than the pressure at which max*f*_o_ was obtained (mean ± SE = 18.73 ± 2) (Z = −3.94, P < 0.001; see Supplementary Table S3 for raw data). This corroborates the expectation that *f*_o_ is positively correlated with Psub^15^, and supports the approach we used, i.e using minimal subglottal pressure in order to obtain a standardized comparison of *f*_o_ (through min*f*_o_) across species.

## Discussion

This study is the first empirical examination of the physical and physiological factors underlying size-frequency allometry across multiple primate species. Using a sample of 11 species for which the length of the laryngeal vocal folds (ranging from 7.46 to 64.4 mm) and size of the entire body (ranging from 30 to 98 cm) was known, we recorded *in vitro* phonation in a setup that allows vocal fold tension to be kept at a minimal level while maintaining precise control over subglottal pressure. While previous conclusions have typically been drawn from averages over a large number of species and/or vocalization samples (e.g. refs^6,7^), our approach has the advantage of investigating acoustic allometry with matching anatomical and vocal production data. This provides an unprecedented opportunity to explore the causal determinants of *f*_o_ with a constrained interpretation of the mechanisms at work in this process.

As predicted by Morton^12^, and echoing more recent findings^6,7^, we found that calls from larger species indeed have lower *f*_o_, as shown by the significant negative min*f*_o_ - body size correlation in our data. In agreement with theoretical predictions^15^, we also found that calls produced with longer vocal folds have a lower min*f*_o_. Additionally, our data show that VFL is the best predictor of the minimum fundamental frequency attainable by phonation of the specimens larynges (Fig. 2a and b). PGLS analyses (that controlled for non-independence of data points due to shared ancestry of species) confirmed these results, as VFL was still, by far, the stronger predictor of min*f*_o_ in these analyses (Fig. 2a and b).

In addition to documenting the moderate strength of the VFL – body size regression (Fig. 1a), these results also illustrate the considerable variability of relative laryngeal size across primate species, independent of body size. This decoupling between larynx size and overall body size can occur because laryngeal growth is not tightly constrained by the rest of the body^5^. The soft cartilaginous structure of the larynx combined with its location, connected loosely in primates via muscles and ligaments to the skull, jaw and sternum, allows its independent growth during development^5^, potentially influenced by hormone levels or other size-independent factors^38^. This peculiar anatomical independence can allow larynx size to be sensitive to various selective pressures that may differ from those acting on body size.

Ecological factors are among the potential selective forces acting on vocalization frequency. For example, species-specific habitat could have fine-tuned laryngeal anatomy by favorably selecting vocalizations produced within a certain frequency range (the ‘acoustic adaptation hypothesis’^39^). Hauser^20^ suggested that the lower frequency range found for *Macaca silenus* could be one such example, as the tropical rainforest home to this species might impair the propagation of higher frequencies^40^. Although our analyses focus on min*f*_o_ rather than complete frequency range, this suggestion is supported by the fact that a species of relatively similar size (*Macaca sylvanus*), inhabiting more open habitats^41^, has a min*f*_o_ 50% higher than that of *M. silenus* (185.85 Hz and 123.81 Hz, respectively). While the propagation of these two frequencies *per se* might not differ much in tropical and open habitats, the apparent predisposition of the *M. sylvanus* larynx to produce higher-pitched vocalizations is worthy of further investigation and environmental propagation experiments.

Second, species-specific socio-ecology also has the potential to influence laryngeal anatomy independently from body size, so that it better suits the requirements of a given species’ vocal communication system^42^. The apes included in this study provide an illustrative case of this possibility: despite being very close in terms of measured body length (94 cm for the female gorilla, 98 cm for the female chimpanzee), and vocal fold length (38.25 mm vs. 35.4 mm, respectively), min*f*_o_ in the chimpanzee was over 3 times higher than that of the gorilla (88.32 Hz and 27.44 Hz, respectively; Table 1). Structural aspects of vocal fold composition differ between these two species^43^, and such histological differences may result from selection for different communicative needs and call usage inherent to these species’ social systems. Chimpanzees live in fission-fusion systems^44^ and vocalize mostly in long-distance communicative contexts using loud, high-frequency pant-hoots^45^. Gorillas, on the other hand, live in more cohesive social groups^46^ and typically vocalize at closer range mostly using low frequency grunts^45,46^. A vocal fold structure suitable for higher-frequency call production in chimpanzees and lower frequency call production in gorillas could thus contribute to explaining why the theoretically-predicted correlation between min*f*_o_ and VFL does not lead to similar observations in these close relatives of humans. Additional histological data would be required to evaluate this hypothesis, focusing for instance on vocal fold elasticity as this parameter has been shown to affect *f*_o_^30^.

**Table 1:** Primate species used in the study, including specimen sex, body size (from anatomical measurements), vocal fold length (estimated from CT-scan measurements) and min*f*_o_ values (from excised larynx experiments); epiglottis position when min*f*_o_ was obtained is also indicated for each species.

Family	Species	Common name	Sex	Body length (cm)	VFL (mm)	Min*f*_o_ (Hz)	Epiglottis position
Atelidae	*Alouatta caraya*	Black howler	F	57	57.76	35.61	Covering
Atelidae	*Alouatta sara*	Bolivian red howler	M	65.5	64.40	27.61	Retracted
Atelidae	*Ateles fusciceps*	Black-headed spider monkey	M	53.5	20.36	81.47	Covering
Hominidae	*Gorilla gorilla*	Western gorilla	F	94	35.40	27.44	Retracted
Cercopithecidae	*Macaca fuscata*	Japanese macaque	F	72.6	17.15	91.49	Covering
Cercopithecidae	*Macaca silenus*	Lion-tailed macaque	F	53	15.70	123.81	Retracted
Cercopithecidae	*Macaca sylvanus*	Barbary macaque	F	59	14.87	185.85	Covering
Hominidae	*Pan troglodytes*	Chimpanzee	F	98	38.25	88.32	Retracted
Cercopithecidae	*Papio hamadryas*	Hamadryas baboon	M	78	25.05	106.98	Covering
Cebidae	*Saimiri sciureus*	Common squirrel monkey	M	30	7.46	658.48	Covering
Lemuridae	*Varecia variegata*	Black-and-white ruffed lemur	F	50	17.86	161.37	Retracted

Finally, sexual selection is an evolutionary force for which there is already some evidence of an influence on laryngeal growth, leading to a decoupling of larynx size from overall body size^6^. Howler monkeys provide one of the most drastic example of hypertrophied vocal apparatus^37,47^ and thus have disproportionately low frequency vocalizations^33,42^. However, males howlers’ larynges and hyoids are enlarged to a much greater extent than those of females^42^. As outlined in a recent study^48^, mating systems appear to strongly influence *f*_o_ dimorphism in anthropoid primates, including humans^49^. Appropriate playback experiments^50^ will be necessary to investigate the effect of acoustic traits in howlers vocalizations that are potentially relevant to sexual partners and/or competitors, as previously done in other species (e.g. refs^51–53^). Size exaggeration often occurs via behavioral/anatomical adaptations affecting formants^6^. However, howler laryngeal hypertrophy affects both vocal fold length and vocal tract morphology (as air sacs fill the enlarged thyro-hyoid apparatus and may act as a resonance chamber^37,54^). This explains the abnormally low *f*_o_ and formants that characterize howler species vocalizations^42^, given that their vocal folds and vocal tracts are considerably larger than those of similarly sized primates (e.g., macaques). In this context, it appears evident that howler vocalizations do not provide honest signals about the size of the caller when making across taxa comparisons. However, similar to red deer vocal tract elongation during roaring^55^, or koala descended larynx for bellowing^19^, howler vocalizations are likely to provide a case of honest signalling when considering vocal production within the species^42^; again, answering this will require further research, combining anatomical investigation and playback of resynthesized signals.

Because our larynges were from dead animals, all oscillations observed were generated by passive airflow. It must be noted that such conditions do not necessarily reproduce the lowest possible *f*_o_s: vocal fold oscillation may in a few cases be induced by active contraction of the vocal fold musculature (the so-called “active” theory of phonation, as apparently applicable to cat purring^56^). Because of limits on the rate of muscular contraction, active phonation is only expected to be possible for *f*_o_s below ~40 Hz^34,57^. An exception is the superfast laryngeal muscles of bats, which are specially adapted to contract up to 180 times a second in some species^58^. Aside from cat purring, however, mammalian phonation is typically generated without periodic muscular contraction at each *f*_o_ period, and instead produced by the airflow passing through the glottis (the myoelastic-aerodynamic theory (MEAD; refs^28,32,59^). The excised larynx methodology applied here illustrates the generality of the MEAD principle, broadening the range of primate species to which it can be applied. For example, our experiments show that the MEAD principle is sufficient to explain the low *f*_o_s of the two howler species we investigated, as we were able to get low frequencies for these species (*A. caraya*: 35.61 Hz; *A. sara*: 25.42 Hz), comparable to those observed in their natural vocalizations^42^, entirely with passive phonation. While not definitely ruling out the possibility of active phonation in our species, this suggests that no active contraction of the laryngeal muscles is required to produce the low-frequency calls typical of howler species.

Based on theoretical predictions and the reasoning outlined in the introduction, we used min*f*_o_ as our standard frequency measure. With our setup, we had no means of controlling whether the higher end of our applied pressure range (on which max*f*_o_, and thus mean*f*_o_, theoretically depend) was physiologically relevant (i.e., matched what the living animal is capable of; pressure measurements, via tracheal catheter, would be necessary to determine this). Min*f*_o_ is therefore the only frequency measure obtained with clear boundary conditions, and thus that can reasonably be assumed to have physiological relevance. Given this, it is intriguing that all *f*_o_ measures (min*f*_o_, mean*f*_o_, max*f*_o_) were significantly negatively related with VFL, and that min*f*_o_ had a weaker correlation with body size than mean*f*_o_ and max*f*_o_ (Supplementary Table S2). This highlights the importance of caution while conducting acoustic allometry research based on non-controlled acoustic data from living animals; it is crucial to avoid false positives by broadly sampling the vocalization types utilized in the analyses.

Two further points support the use of min*f*_o_ and minimal Psub in future analyses of this sort: 1) the fit of regressions between VFL and max*f*_o_ (as well as VFL and mean*f*_o_) were not as good as those between VFL and min*f*_o_, (Supplementary Table S2) and 2) Psub at min*f*_o_ was significantly lower than Psub at max*f*_o_. In-depth investigation of the Psub-*f*_o_ relationship would be valuable, but is beyond the scope of this study. Such investigations require extreme caution, as preservation of the biomechanical properties of vocal fold tissue (e.g. viscoelasticity) may be affected by the time elapsed between death and specimen freezing, which in turn may have affected PTP^60^ and potentially altered the resulting min*f*_o_. While we acknowledge this potential limitation in our protocol, we point out (1) the difficulty of gathering such a collection of specimens: for many species it is rare to be able to acquire even a single specimen within a time span of a decade or more (e.g. apes or howler monkeys) and (2) that given the systematic variation observed in the relationship between VFL and min*f*_o_, the data collected here offer unique insights into the physical determinant of vocal frequency and the decoupling between larynx and body size in a representative sample of primates. We would also like to call attention to the fact that, although our selection of larynges was based on specimen availability and thus included either male or female larynges, the large range of body sizes observed across the species considered here should limit the impact of any potential sexual dimorphism on our results. However, given the more limited size variation, along with the potential for size dimorphism, within a species, future studies investigating the decoupling between larynx and body size at an intra-specific level should ensure the selection of larynges from the same sex.

Ultimately, by adopting a novel allometric approach, our study both confirms the theoretical prediction that vocal fold length is a main determinant of *f*_o_, and provides evidence that laryngeal growth is not tightly constrained by overall body size (at least in the primate species investigated here). Our results call attention to the considerable anatomical variation across species that can be observed in primate vocal production systems, most of which has barely been investigated. As illustrated by the hypotheses we raise, this variability offers great potential for future in-depth studies of how various selective pressures may have driven diversity in vocal production and anatomy in primates and other mammals. Further work using excised larynx systems like that described here are critical to improving our understanding of mammalian vocal production mechanisms, and thus of the functions of mammal vocal communication viewed from an evolutionary perspective.

## Methods

### Data collection

#### Anatomical specimens

**Figure 3:**
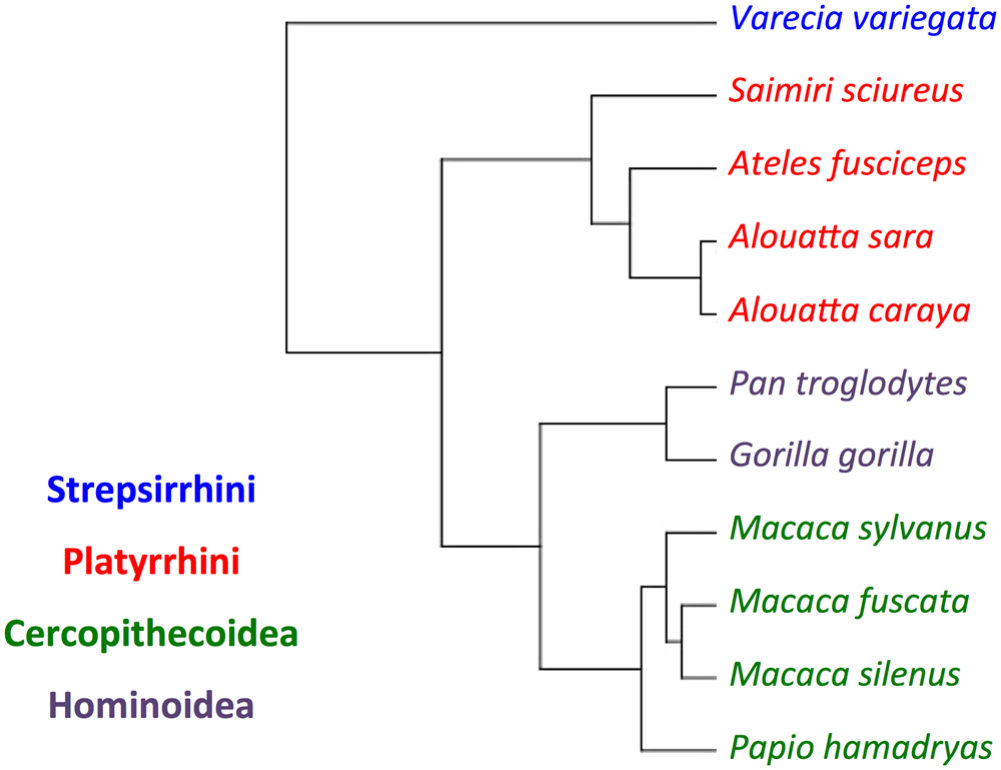
Consensus tree of phylogenetic distances among the species examined in this study. Tree based on a combination of 2 to 16 DNA sequences among 11 mitochondrial and 6 autosomal genes retrieved from mitochondrial and autosomal data available from *10kTrees*^71^, version 3 at http://10ktrees.nunn-lab.org/project.html; see Supplementary Table S1.

As part of the specimen acquisition program at the Department of Natural Sciences, National Museums Scotland, the remains of deceased European zoo animals are regularly collected and processed. Our larynges came from these zoo specimens and all samples came from animals that had died of natural causes. For each individual, body size was measured as the distance between the ischium of the pelvis and the top of the skull (head-body length, without tail). Body length was preferred over body weight because: (1) obesity is a potential problem in zoo animals^61^; (2) bodies can dehydrate once deceased^62^ making post mortem weight dependent on measurement delay; and (3) weight data could not be obtained for some of our specimens. Larynges from the cadavers of 11 individuals, each of a different primate species, were excised, frozen and stored at −20° Celsius at the National Museums Scotland before being shipped to the Department of Cognitive Biology, University of Vienna (see Supplementary Text for additional information on the freezing method). Each larynx was then thawed, cleaned, inspected, photographed and measured in preparation for X-ray CT scanning, after which they were refrozen and stored at −20 °C. The primates used for this study were chosen to represent a wide range of body sizes and phylogenetic diversity (Fig. 3 and Table 1).

### CT scans

Two procedures were applied, depending on the size of the specimen: the larynx of the smallest species (squirrel monkey, *Saimiri sciureus*,) was scanned using micro CT, while ordinary CT was used for the other 10 larynges. All CT scans were performed at the University of Veterinary Medicine Vienna. Macro CT scans were made using a Siemens SOMATOM Emotion helical CT-scanner (Siemens AG, Munich, Germany), and the micro-CT scan was made using an Xradia microXCT-400 (0.4x lens; Carl Zeiss X-ray Microscopy, Pleasanton, CA). For macro-CT scans, specimens were positioned in ventral recumbancy on X-ray-transparent styrofoam plates and scanned frozen. Scanning parameters were adjusted to specimen size, using 110–130 kV source voltage and 80–110 mA beam intensity. Reconstructed image slices measured 512 × 512 pixels. Depending on larynx size, the dimensions of reconstructed voxels varied between 238–340 µm^2^ in the xy plane and 200–500 µm in the z plane. Due to its small size and longer scanning time, the *Saimiri* specimen was thawed prior to micro-CT scanning and mounted vertically inside a sealed Falcon tube, the bottom of which was partially filled with phosphate-buffered saline to prevent dehydration. The specimen was scanned at 40 keV source voltage and 200 µA beam intensity. Reconstructed slices measured 512 × 512 pixels and the voxel resolution of reconstructed volumes was 35 µm^3^.

### Excised larynx experiments

A detailed description of the setup used in this study has been given elsewhere^63^. Before use in excised larynx experiments (Table 1) each specimen was thawed, then prepared by removing excess tissue and tracheal rings, before being mounted on a vertical subglottic tube. The tube diameter was adjusted to match specimen size such that an airtight seal was formed with the trachea. Larynx stability and support were ensured using a combination of adjustable plastic support structures (made of LEGO blocks, Billund, Denmark) and custom-made 3D-printed plastic mounts placed on the left lateral, right lateral and anteriorly sides of the larynx.

Phonation was obtained by passing a controlled flow of warm (~37 °C) humid (100%) air through the mounted larynx. Vocal folds were adducted using 2 manually controlled micromanipulators (Warzhauser MM33, Tamm, Germany) mounted on a tilting platform. For standardization purposes, the degree of adduction was fixed when phonation could be reliably induced with minimal airflow and tension on the vocal folds, and attained a steady phonation (assessed by ear and via examination of the electroglottographic (EGG) signal during the experiment). Custom-made copper EGG electrodes were placed on both sides of the thyroid cartilage, at the level of the vocal folds, for an optimal recording of vocal fold vibrations. Psub was controlled using “ELLApp” software (created in Python by CTH). Acoustic, EGG and sound intensity were recorded using a DPA 4061 omnidirectional microphone (positioned at a variable but known distance from the vocal folds), a Glottal Enterprises EG 2-1000 two-channel electroglottograph (lower cutoff-frequency 2 Hz) and an NL-52 RION sound pressure level-meter (located 30 cm from the vocal folds; settings ‘fast acquisition’ and ‘dB C’ weighting), respectively. All signals were acquired, synchronized and stored within ELLApp.

Phonation and data acquisition followed an adjustable computer-controlled sequence. Pressure sweeps were applied to each excised larynx, consisting of a slow linear increase in Psub followed by a slow linear decrease of the same duration; the lowest Psub value was set just below the PTP, and the highest value varied with specimen size. Each larynx was exposed to 4–8 pressure sweeps, 2–4 with the epiglottis covering the airway and 2–4 with the epiglottis retracted. The aim of epiglottis manipulation was to evaluate whether a source-filter interaction (so-called “feedback” system, refs^5,64^) exists between the vibrating vocal folds and what is left of the vocal tract in our setup, i.e. the space between the glottis and the epiglottis. The number of sweeps was chosen to allow us to evaluate repeatability of acoustic production while avoiding damage or drying of the sound source. Throughout the experiments, larynges were kept moist using a spray-bottle containing saline solution (0.9% NaCl).

### Data analysis

#### Anatomical measurements: CT scans

**Figure 4:**
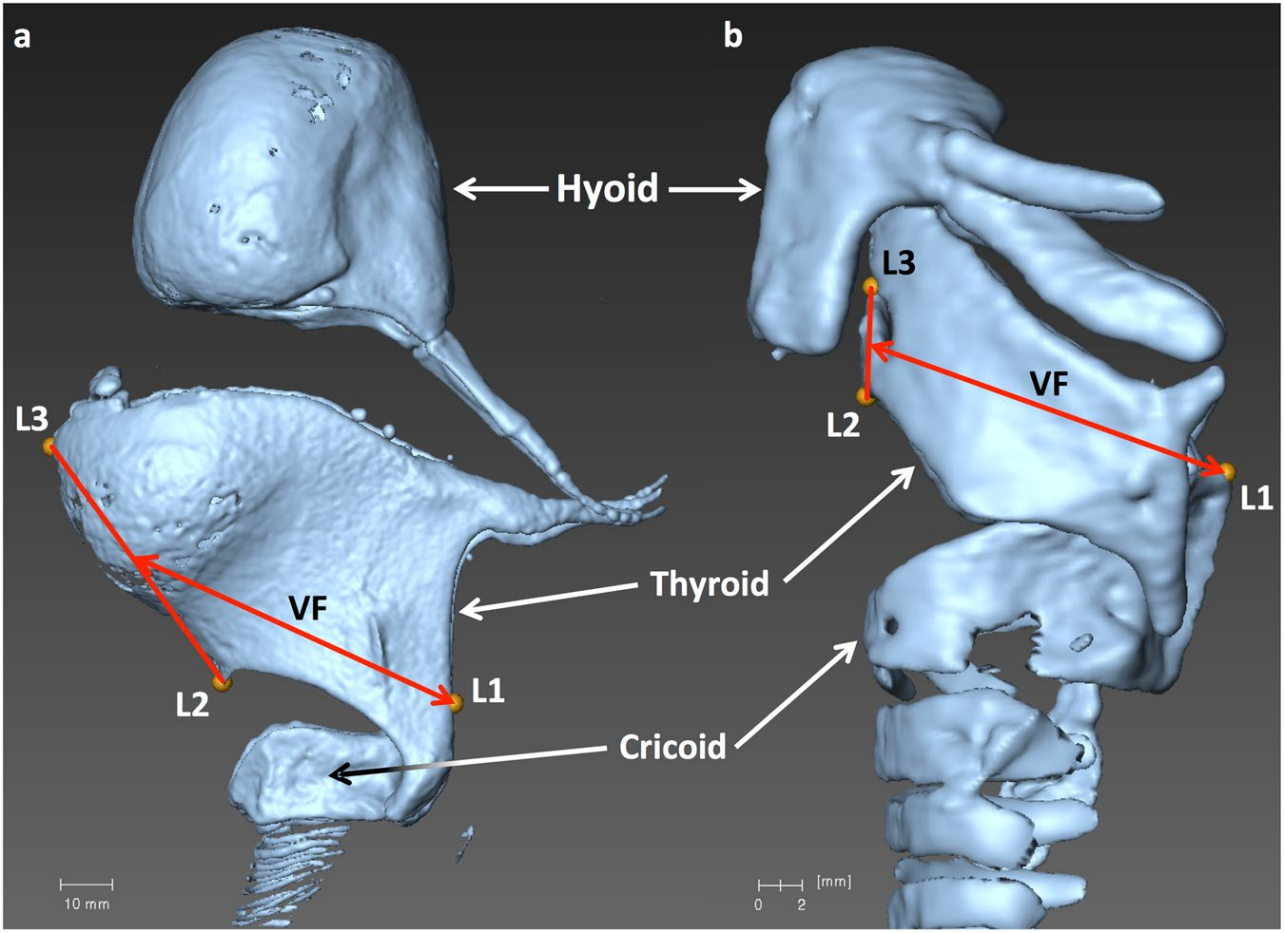
Isosurface of large and small laryngeal specimens. Panel a (*Alouatta sara)* and panel b (*Macaca fuscata*) show the homologous landmarks used to establish the vocal fold length proxy. L1: Dorsal apical cricoid; L2: Ventral basal thyroid; L3: Ventral apical thyroid. VF: segment used as the skeletal proxy for vocal fold length (not to scale).

Both macro and micro CT data were analyzed using AMIRA software (version 5.6.0). Along their length, the vocal folds are composed of a membranous and a cartilaginous section^65^. Soft tissue geometry is difficult to visualize in CT, and direct measurement of VFL would have required tissue destruction and perhaps histology to be accurately determined. Thus our aim was to obtain clear 3D visualization of the laryngeal cartilages (hyoid bone, thyroid, cricoid and arytenoid cartilages^66^) to estimate the total vocal fold length (membranous + cartilaginous length). After creating an isosurface model of these cartilages, VFL was estimated based on measurement of homologous landmarks placed at the intersection of the mid-sagittal plane and the cricoid and thyroid cartilages (Fig. 4). The most dorsal point for our vocal fold estimate was placed at the apex of the cricoid cartilage on the midline. The most ventral possible attachment point of the vocal folds was defined as the midpoint between the apex and the base of the thyroid cartilage (as the thyroid attachment of the vocal fold could not be consistently visualized from the CT data).

### Signal analysis

The analysis of *f*_o_ from acquired signals was conducted using the autocorrelation function in Praat^67^, and in ELLApp. After synchronization of the various input signals in ELLApp, EGG signals were annotated in Praat and *f*_o_ was extracted with appropriately adjusted settings (Praat function ‘To Pitch (ac)…’, creating a Praat ‘PitchTier’ object; see Supplementary Text for details). Settings were adjusted both relying on visual inspection of the spectrograms (to identify and exclude non-periodic regimes; time step was automatically computed as 0.75/ *pitch_floor*, which varied between 20 Hz and 620 Hz depending on the pressure sweep and species analyzed) and of the waveform (to further identify and exclude ambiguous nonlinear phenomena like subharmonics)^68,69^. The raw *f*_o_ data produced by Praat (termed “PitchTier” in Praat) was then edited manually in order to exclude any pitch-tracking errors made by the automated *f*_o_ extraction algorithm. We excluded all parts of the signals characterized by non-periodic oscillation of the vocal folds (with periodicity defined as a minimum of 10 regular consecutive vocal fold oscillatory cycles), as well as those regions where Praat’s automated calculation did not correspond to the lowest partial visible in the spectrogram and/or the main oscillation in the waveform. The minimum, maximum and mean *f*_o_ (min*f*_o_, max*f*_o_ and mean*f*_o_, respectively) were queried from this corrected pitch object based on the remaining annotated sections, using the Praat ‘Get minimum…’, ‘Get maximum…’ and ‘Get mean…’ functions.

Using the calibrated data from ELLApp, we also extracted the Psub values obtained at min*f*_o_ and max*f*_o_ to evaluate the effect of Psub on *f*_o_ and verify our approach of using minimal tension and Psub to attain min*f*_o_.

### Statistics

Following assessment of data normality using Shapiro-Wilk tests, body size, VFL and min*f*_o_ were log-transformed (base 10; see raw data values Table 1) and the following OLS linear regressions (i.e. standard linear regression models) were computed: log min*f*_o_ vs. log body size, log min*f*_o_ vs. log VFL, and log body size vs. log VFL. Additionally, due to the potential influence of species relatedness, PGLS regressions (which accounts for the potential non-independence of data points due to shared phylogenetic history; see ref.^70^) were also computed on the same set of variables, controlling for the potential effects of phylogenetic covariance^70^, using the consensus phylogenetic tree shown in Fig. 3 (created using *10kTrees*^71^, version 3 (http://10ktrees.nunn-lab.org/project.html)).

In order to evaluate the validity of focusing on min*f*_o_, for comparative purposes we conducted the same set of analyzes on max*f*_o_ and mean*f*_o_. Because howler monkeys are clear anatomical outliers due to their greatly enlarged vocal apparatus^37,42,47^, we also conducted the same set of analyses omitting the two howler specimens to evaluate whether our results were solely driven by these extreme cases. Finally, ‘Sex’ and ‘Epiglottis’ variables were included either alone or together in preliminary linear and PGLS models, but showed no significant effect in any of all the possible combinations. These two variables were thus omitted from subsequent analyses.

To evaluate the effect of subglottal pressure on *f*_o_, Psub values at min*f*_o_ and max*f*_o_ were compared using a Wilcoxon signed-rank test.

All statistics and computations were done in R^72^ using the ‘lm’ function for OLS regressions and the ‘pgls’ function (‘caper’ package) for PGLS regressions^73^. Two-tailed P-values are reported and significance level is set at 0.05.

## Electronic supplementary material

Supplementary information
