# Publisher Correction: Acoustic allometry revisited: morphological determinants of fundamental frequency in primate vocal production

**DOI:** 10.1038/s41598-017-18707-x

**Published:** 2018-01-15

**Authors:** Maxime Garcia, Christian T. Herbst, Daniel L. Bowling, Jacob C. Dunn, W. Tecumseh Fitch

**Affiliations:** 1 Department of Cognitive Biology, University of Vienna, Althanstrasse 14, 1090 Vienna, Austria; 2 ENES Lab, Université Lyon/Saint-Etienne, NEURO-PSI, CNRS UMR 9197 Saint-Etienne, France; 3 Division of Biological Anthropology, University of Cambridge, Pembroke Street, Cambridge, CB2 3QG UK; 4 Animal and Environment Research Group, Anglia Ruskin University, East Road, Cambridge, CB1 1PT UK

Correction to: *Scientific Reports* 10.1038/s41598-017-11000-x, published online 05 September 2017

This Article contains errors in Equation 1.

$${f}_{o}=\,\frac{1}{2L}\sqrt{\frac{\sigma }{2L}}[{\rm{Hz}}]$$

should read:

$${f}_{o}=\,\frac{1}{2L}\sqrt{\frac{\sigma }{\rho }}[{\rm{Hz}}]$$

