# Publisher Correction: Acoustic allometry revisited: morphological determinants of fundamental frequency in primate vocal production

**DOI:** 10.1038/s41598-018-22366-x

**Published:** 2018-03-06

**Authors:** Maxime Garcia, Christian T. Herbst, Daniel L. Bowling, Jacob C. Dunn, W. Tecumseh Fitch

**Affiliations:** 1 Department of Cognitive Biology, University of Vienna, Althanstrasse 14, 1090 Vienna, Austria; 2 ENES Lab, Université Lyon/Saint-Etienne, NEURO-PSI, CNRS UMR 9197 Saint-Etienne, France; 3 Division of Biological Anthropology, University of Cambridge, Pembroke Street, Cambridge, CB2 3QG UK; 4 Animal and Environment Research Group, Anglia Ruskin University, East Road, Cambridge, CB1 1PT UK

Correction to: *Scientific Reports* 10.1038/s41598-017-11000-x, published online 05 September 2017

An error occurred after publication resulting in the inadvertent omission of the Article and Volume numbers from the PDF version of this Article. This error has now been corrected in the PDF version of the Article; the HTML version was correct from the time of publication.

