# Supplementary material for: Acoustic allometry revisited: morphological determinants of fundamental frequency in primate vocal production
Source: Sci Rep. 2017 Sep 5;7:10450. doi: 10.1038/s41598-017-11000-x (PMC5585385; doi:10.1038/s41598-017-11000-x)
Supplement: Supplementary file 1 — Supplementary information [file 41598_2017_11000_MOESM1_ESM.pdf]

Acoustic allometry revisited: morphological determinants of fundamental frequency in primate vocal production

Maxime Garcia, Christian T. Herbst, Daniel L. Bowling, Jacob C. Dunn and W. Tecumseh Fitch.

SUPPLEMENTARY INFORMATION

Supplementary Table

Supplementary Table S1: Availability matrix for the sequences used to create a consensus primate phylogenetic tree. Data retrieved from *http://10ktrees.nunn-lab.org/project.html.* Full names and type of gene sequences are indicated below Sup. Tab. 1.

Species	12S rRNA	16S rRNA	CCR5	COX1	COX2	COX3	CYTB	IRBP	MC1R	ND1	ND3	ND4	ND4I	ND5	PRP	SRY	TSPY	Total No. of genes
Alouatta caraya			X		X		X		X									4
Alouatta sara							X		X									2
Ateles fusciceps	X	X			X													3
Gorilla gorilla (gorilla)	X	X	X	X	X	X	X			X	X	X	X	X				12
Macaca fuscata	X	X	X	X	X			X	X			X			X	X	X	11
Macaca silenus	X	X		X	X	X	X		X							X	X	9
Macaca sylvanus	X	X		X	X	X	X		X	X	X	X	X	X	X	X	X	15
Pan troglodytes (troglodytes)	X	X	X	X	X	X	X			X	X	X	X	X				12
Papio hamadryas	X	X	X	X	X	X	X		X	X	X	X	X	X	X	X	X	16
Saimiri sciureus	X	X	X	X	X	X	X	X	X	X	X	X	X	X	X	X		16
Varecia variegata (variegate)	X	X		X	X	X	X	X	X	X	X	X	X	X	X			14

12S rRNA:	12S ribosomal rRNA - TYPE: MITOCHONDRIAL
16S rRNA:	16S ribosomal rRNA - TYPE: MITOCHONDRIAL
CCR5:	C-C chemokine receptor type 5 - TYPE: AUTOSOMAL
COX1:	Cytochrome c oxidase subunit I - TYPE: MITOCHONDRIAL 119
COX2:	Cytochrome c oxidase subunit II - TYPE: MITOCHONDRIAL 157
COX3:	Cytochrome c oxidase subunit III - TYPE: MITOCHONDRIAL 63
CYTB:	Cytochrome B - TYPE: MITOCHONDRIAL 228
IRBP:	Interphotoreceptor retinoid-binding protein - TYPE: AUTOSOMAL
MC1R:	Melanocortin 1 receptor - TYPE: AUTOSOMAL
ND1:	NADH dehydrogenase subunit 1 - TYPE: MITOCHONDRIAL 66
ND3:	NADH dehydrogenase subunit 3 - TYPE: MITOCHONDRIAL 141
ND4:	NADH dehydrogenase subunit 4 - TYPE: MITOCHONDRIAL 168
ND4L:	NADH dehydrogenase subunit 4L - TYPE: MITOCHONDRIAL 152
ND5:	NADH dehydrogenase subunit 5 - TYPE: MITOCHONDRIAL 74
PRP:	Major prion protein (encoded by the PRNP gene) - TYPE: AUTOSOMAL
SRY:	Sex-determining Region Y - TYPE: AUTOSOMAL
TSPY:	Testis-specific Y-encoded protein 1 (encoded by the TSPY1 gene) - TYPE: AUTOSOMAL

Supplementary Table S2: Results from the PGLS and linear regression models, with and without including the two howler species in the analyses, fitted to explain variance in 'log minF0' (top panel), 'log meanF0' (middle panel) and 'log maxF0' (bottom panel) with 'log body size' and 'log VFL'.

**Regression model (x vs. y)**	**Method**	**Howlers**	**N**	**β**	**SE**	**λ**	**t**	**p**	**r^2**
log(body size) vs. log(minf_o)	PGLS	Yes	11	-2.4	0.69	0.59	-3.48	0.007	0.53
log(body size) vs. log(minf_o)	OLS	Yes	11	-1.95	0.69	-	-2.82	0.02	0.41
log(VFL) vs. log(minf_o)	PGLS	Yes	11	-1.31	0.2	0.00	-6.52	< 0.001	0.81
log(VFL) vs. log(minf_o)	OLS	Yes	11	-1.31	0.2	-	-6.52	< 0.001	0.81
log(body size) vs. log(minf_o)	PGLS	No	9	-1.95	0.47	0.00	-4.11	0.004	0.67
log(body size) vs. log(minf_o)	OLS	No	9	-1.95	0.47	-	-4.11	0.004	0.67
log(VFL) vs. log(minf_o)	PGLS	No	9	-1.48	0.32	0.00	-4.64	0.002	0.72
log(VFL) vs. log(minf_o)	OLS	No	9	-1.48	0.32	-	-4.64	0.002	0.72

**Regression model (x vs. y)**	**Method**	**Howlers**	**N**	**β**	**SE**	**λ**	**t**	**p**	**r^2**
log(body size) vs. log(meanf_o)	PGLS	Yes	11	-2.27	0.51	1.00	-4.46	0.002	0.65
log(body size) vs. log(meanf_o)	OLS	Yes	11	-1.69	0.55	-	-3.07	0.01	0.46
log(VFL) vs. log(meanf_o)	PGLS	Yes	11	-1.03	0.21	0.00	-4.88	< 0.001	0.7
log(VFL) vs. log(meanf_o)	OLS	Yes	11	-1.03	0.21	-	-4.88	< 0.001	0.7
log(body size) vs. log(meanf_o)	PGLS	No	9	-1.68	0.47	0.00	-3.6	0.009	0.6
log(body size) vs. log(meanf_o)	OLS	No	9	-1.68	0.47	-	-3.6	0.009	0.6
log(VFL) vs. log(meanf_o)	PGLS	No	9	-1.3	0.31	0.00	-4.18	0.004	0.67
log(VFL) vs. log(meanf_o)	OLS	No	9	-1.3	0.31	-	-4.18	0.004	0.67

**Regression model (x vs. y)**	**Method**	**Howlers**	**N**	**β**	**SE**	**λ**	**t**	**p**	**r^2**
log(body size) vs. log(maxf_o)	PGLS	Yes	11	-2.05	0.55	0.66	-3.7	0.005	0.56
log(body size) vs. log(maxf_o)	OLS	Yes	11	-1.62	0.55	-	-2.93	0.02	0.43
log(VFL) vs. log(maxf_o)	PGLS	Yes	11	-1.04	0.19	0.00	-5.45	< 0.001	0.74
log(VFL) vs. log(maxf_o)	OLS	Yes	11	-1.04	0.19	-	-5.45	< 0.001	0.74
log(body size) vs. log(maxf_o)	PGLS	No	9	-1.62	0.48	0.00	-3.36	0.01	0.56
log(body size) vs. log(maxf_o)	OLS	No	9	-1.62	0.48	-	-3.36	0.01	0.56
log(VFL) vs. log(maxf_o)	PGLS	No	9	-1.34	0.27	0.00	-5.06	0.001	0.75
log(VFL) vs. log(maxf_o)	OLS	No	9	-1.34	0.27	-	-5.06	0.001	0.75

Supplementary Table S3: Raw data of pressure values obtained respectively at MinF0 and MaxF0 for all pressure sweeps applied across experiments.

Species	Recording ID	Sweep Nb	Epiglottis position	MinF0	Psub at minF0	MaxF0	Psub at maxF0
Papio hamadryas	3362	1	out	123.23	18.1	167.79	11.253
Papio hamadryas	3362	2	out	129.46	15.81	168.75	10.97
Papio hamadryas	3363	1	above	106.98	22.65	259.25	8.15
Papio hamadryas	3363	2	above	108.37	22.05	232.9	12.25
Alouatta caraya	3392	1	out	44.44	10.07	128.95	44.01
Alouatta caraya	3392	2	out	44.7	8.61	115.22	43.96
Alouatta caraya	3393	1	above	41.27	10.23	112.54	29.66
Alouatta caraya	3393	2	above	35.61	8.75	113.62	42.49
Macaca sylvanus	3335	1	above	185.85	20.12	359.86	9.56
Macaca sylvanus	3335	2	above	240.48	28.38	429.29	13.23
Macaca sylvanus	3336	1	out	335.22	27.59	525.62	23.87
Macaca sylvanus	3336	2	out	328.04	26.9	515.45	21.5
Macaca silenus	3342	1	above	142.18	10.31	230.67	28.77
Macaca silenus	3342	2	above	143.96	10.35	219.61	26.18
Macaca silenus	3343	1	out	123.81	10.38	219.01	25.88
Macaca silenus	3343	2	out	125.22	10.78	218.07	7.22
Alouatta seniculus	3429	1	above	28.64	4.22	92.94	21.77
Alouatta seniculus	3429	2	above	46.58	8.37	91.08	21.24
Alouatta seniculus	3432	1	out	27.61	4.76	96.34	23.02
Alouatta seniculus	3432	2	out	31.1	5.37	93.75	22.61
Alouatta seniculus	3432	3	out	32.11	3.31	94.42	21.88
Macaca fuscata	3466	1	above	91.49	6.26	170.64	3.74
Macaca fuscata	3466	2	above	99.23	7.34	164.37	31.37
Macaca fuscata	3467	1	out	147.99	7.12	226.68	3.35
Macaca fuscata	3467	2	out	146.99	8.93	224.28	3.89
Saimiri sciureus	3514	1	out	939.3	19.8	1350.82	33.07
Saimiri sciureus	3514	2	out	943.24	14.59	1274.1	39.71
Saimiri sciureus	3514	3	out	970.35	15.43	1296.74	37.33
Saimiri sciureus	3515	1	above	658.48	7.02	920	50.23
Saimiri sciureus	3515	2	above	663.47	6.62	943.25	50.63
Saimiri sciureus	3515	3	above	683.98	6.28	1071.21	50.37
Saimiri sciureus	3515	4	above	700.56	6.12	1072.26	50.61
Varecia variegata	3775	1	above	178.23	7.06	240.48	2.79
Varecia variegata	3775	2	above	196.11	5.84	235.47	2.59
Varecia variegata	3775	3	above	196.07	5.39	234.48	2.52
Varecia variegata	3776	1	out	161.37	7.59	249.21	2.03
Varecia variegata	3776	2	out	181.73	12.75	246.15	1.96
Varecia variegata	3776	3	out	181.98	13.62	226.72	9.34
Pan troglodytes	3579	1	above	92.77	1.73	115.31	8.42
Pan troglodytes	3579	2	above	91.88	1.54	114.57	8.18
Pan troglodytes	3579	3	above	90.04	1.52	113.6	8.12
Pan troglodytes	3580	1	out	90.23	1.66	112.73	7.8
Pan troglodytes	3580	2	out	88.83	1.62	110.58	8.29
Pan troglodytes	3580	3	out	88.32	1.67	109.1	7.93
Gorilla gorilla	3602	1	above	85.17	4.5	153.1	14.24
Gorilla gorilla	3602	2	above	79.9	4.52	153.11	14.02
Gorilla gorilla	3602	3	above	86.13	4.81	153.85	13.86
Gorilla gorilla	3603	1	out	45.72	3.71	59.86	2.79
Gorilla gorilla	3603	2	out	27.71	2.29	111.23	7.84
Gorilla gorilla	3603	3	out	27.44	2.16	109.29	7.55
Ateles fusciceps	3641	1	above	83.16	7.86	115.12	13.67
Ateles fusciceps	3641	2	above	81.47	7.89	120.39	13.34
Ateles fusciceps	3641	3	out	81.99	7.25	114.05	11.93
Ateles fusciceps	3641	4	out	85.4	7.07	147.77	18.24

Supplementary Text

Methodological aspects

Freezing process

Slow freezing has been suggested to alter more severely the biomechanical integrity of the vocal folds than quick freezing (using liquid nitrogen) [1]. While we acknowledge these results, in the laryngeal specimens used in this study we believe the influence of the freezing process to be negligible in regards to the question investigated here, for several reasons. First, the slow freezing process was used consistently and any effect at the cellular level should apply equally to all larynges, allowing the comparative approach followed in this study. Second, the frequency range examined to assess the effect of the freezing process (0.01-15 Hz; [1]) is well below the range of minF0 (25.42-658.48 Hz) documented by our data. Third, vocal fold integrity has been examined with canine specimens, whose structural composition differs from that of primates examined to date (the latter being seemingly closer to that of humans [2,3]. Finally, in humans the variation in vocal fold elasticity and viscosity resulting from differential freezing processes seems comparable to (or smaller than) the variation found between individuals of different genders or age classes [1,4], and therefore do not appear to have the potential to significantly affect our observations.

Praat processing

Creating Praat 'PitchTier' objects was made after thorough inspection of the spectrograms (as described in the main text) using file-specific extraction settings. 'Pitch floor' ranged from 20 Hz to 620 Hz, 'pitch ceiling' from 200 Hz to 1500 Hz, 'silence threshold' was set at 0.03 for all recordings, 'voicing threshold' ranged from 0.05 to 0.45, 'octave cost' from 0.01 to 0.05, 'voice/unvoiced cost' from 0.14 to 0.3, and 'octave-jump cost' was set at 0.35 for all recordings.

References:

1 Chan, R. W. & Titze, I. R. Effect of Postmortem Changes and Freezing on the Viscoelastic Properties of Vocal Fold Tissues. *Ann. Biomed. Eng.* **31**, 482-491, doi:10.1114/1.1561287 (2003).
2 Kurita, S., Nagata, K. & Hirano, M. in *Vocal fold physiology: Contemporary research and clinical issues* (eds D. M. Bless & J. H. Abbs) 3-21 (College Hill, 1983).
3 Riede, T. Elasticity and stress relaxation of rhesus monkey (*Macaca mulatta*) vocal folds. *J. Exp. Biol.* **213**, 2924-2932, doi:10.1242/jeb.044404 (2010).
4 Chan, R. W. & Titze, I. R. Viscoelastic shear properties of human vocal fold mucosa: measurement methodology and empirical results. *J. Acoust. Soc. Am.* **106**, 2008-2021 (1999).
